# Behavioral impairments in animal models for zinc deficiency

**DOI:** 10.3389/fnbeh.2014.00443

**Published:** 2015-01-06

**Authors:** Simone Hagmeyer, Jasmin Carmen Haderspeck, Andreas Martin Grabrucker

**Affiliations:** ^1^WG Molecular Analysis of Synaptopathies, Neurology Department, Neurocenter of Ulm UniversityUlm, Germany; ^2^Institute for Anatomy and Cell Biology, Ulm UniversityUlm, Germany

**Keywords:** Zn, Zn2+, ZnT3, MT-3, brain, plasticity, learning, ZIP

## Abstract

Apart from teratogenic and pathological effects of zinc deficiency such as the occurrence of skin lesions, anorexia, growth retardation, depressed wound healing, altered immune function, impaired night vision, and alterations in taste and smell acuity, characteristic behavioral changes in animal models and human patients suffering from zinc deficiency have been observed. Given that it is estimated that about 17% of the worldwide population are at risk for zinc deficiency and that zinc deficiency is associated with a variety of brain disorders and disease states in humans, it is of major interest to investigate, how these behavioral changes will affect the individual and a putative course of a disease. Thus, here, we provide a state of the art overview about the behavioral phenotypes observed in various models of zinc deficiency, among them environmentally produced zinc deficient animals as well as animal models based on a genetic alteration of a particular zinc homeostasis gene. Finally, we compare the behavioral phenotypes to the human condition of mild to severe zinc deficiency and provide a model, how zinc deficiency that is associated with many neurodegenerative and neuropsychological disorders might modify the disease pathologies.

## Introduction

Currently, an estimated 17.3% of the global population is at risk of developing zinc deficiency (Wessells and Brown, 2012) and a prevalence of inadequate zinc intake was estimated with a range from 7.5% in high-income regions to 30% in South Asia (Wessells and Brown, 2012). Zinc is an essential trace metal in the human body that contains about 2 g zinc stored mostly in muscle and bone tissue. However, the zinc content in the brain is surprisingly high. There, zinc is one of the most abundant trace metals and is enriched at the pre-synaptic compartment of nerve cells stored in specific vesicles together with the neurotransmitter glutamate, but also acts at the post-synapse (Bitanihirwe and Cunningham, 2009). Zinc is able to influence synaptic plasticity (Xie and Smart, 1994; Lu et al., 2000), to regulate post-synaptic proteins (Grabrucker, 2014) and has an important role in the formation and maintenance of the structure of the post-synaptic density (PSD), a network of proteins that links the neurotransmitter receptors to downstream signaling components and to the cytoskeleton (Jan et al., 2002; Grabrucker et al., 2011). Additionally, more than 300 enzymes are zinc-dependent, many of them expressed in the central nervous system (CNS). It is thus not surprising that zinc deficiency, among other pathologies, leads to neuronal dysfunction and in turn to characteristic neurobehavioral changes.

There is a broad spectrum of physiological signs of zinc deficiency given that zinc is involved in a great number of biochemical processes. Most commonly, zinc deficiency is associated with skin lesions, such as seen in *Acrodermatitis enteropathica*, an autosomal recessive metabolic disorder affecting the uptake of zinc. Additionally, growth retardation and hypogonadism in males were among the first and frequently reported clinical signs in zinc deficient patients (Prasad, 2013). However, apart from several other symptoms caused by zinc deficiency, such as poor appetite, delayed wound healing, cell-mediated immune dysfunction, and abnormal neurosensory changes (Prasad, 2013), behavioral alterations have been consistently reported. Zinc deficiency may result in depression, emotional instability, increased anxiety and aggression, irritability and deficits in social behavior. Additionally, or as consequence of some factors discussed above, impaired memory and capacity to learn may occur.

Zinc deficiency is associated with multiple disorders. In particular, patients suffering from neurological disorders often show zinc deficiencies. For example, low serum zinc levels have been reported in Autism Spectrum Disorders (ASD), Attention deficit hyperactivity disorder (ADHD), Mood Disorders, such as depression, Schizophrenia (SCZ), and Spinocerebellar ataxia type 2 (Pfaender and Grabrucker, 2014). Furthermore, zinc deficiency has been observed in disorders of the gastro-intestinal (GI) tract, such as malabsorption syndrome, Crohn’s disease, regional ileitis and steatorrhea, as well as liver disease, renal diseases, alcoholism, and sickle cell disease (Prasad, 2013). It is therefore necessary to understand how zinc deficiency will influence the initiation and/or progression of such a disorder and modify the disease phenotype. Here, we will focus especially on the behavioral alterations that can be observed in zinc deficient subjects.

Given that targeted manipulation of zinc levels in humans is hard to achieve and ethically unacceptable, in the past, various animal models for zinc deficiency have been created and analyzed. Zinc deficiency can be readily produced by dietary zinc restriction using special food. In rodents fed with a zinc deficient diet, signs of zinc deficiency occur very soon. For example, a typical reduction in food intake is observed within approximately 3 days (Evans et al., 2004). At the same time, serum zinc levels are decreased (Ohinata et al., 2009). However, zinc levels in the brain are affected only after chronic zinc deficiency. For example, 4-week zinc deprivation decreases hippocampal zinc concentrations in rats (Takeda and Tamano, 2009). In rodents, a diet that induced marginal/mild zinc deprivation contains 10 μg Zn/g. A zinc content of 5–7 μg/g is associated with moderate deprivation, and of <1–2 μg/g with severe deprivation (Golub et al., 1995). In monkeys, a marginal deprivation occurs at a zinc content of 4 μg/g, moderate deprivation at 2 μg/g and severe deprivation at <1 μg/g (with 50 μg Zn/g considered adequate) (Keen et al., 1993).

Maternal zinc deficiency (prenatal zinc deficiency) produces effects ranging from growth retardation and teratogenesis to embryo/fetal death. Additionally, postnatal complications of maternal zinc deficiency such as neurobehavioral and immunological abnormalities can occur. On the other hand, postnatal zinc deficiency in adult life may cause specific and distinct effects itself. Therefore, it is important to consider the different time-points of zinc deprivation, when investigating the effects of zinc deficiency.

Since the last 50 years, zinc, from an ignored mineral, has now become so important that zinc deficiency is recognized as a major worldwide public health problem. In fact, zinc deficiency has been estimated to cause more than 450,000 deaths in children under the age of 5 (Fischer Walker et al., 2009) and about 800,000 deaths (about 1.5% of all deaths) (Nriagu, 2007) annually worldwide and is responsible for about 20% of perinatal mortality. Mild zinc deficiency is especially common in infants, children, women, and elderly people because of either high nutrient requirements or compromised digestion and absorption and contributes to impaired physical and neuropsychological function (Hambidge, 2000). Especially behavioral alterations may pose a so far underestimated effect of zinc deficiency and can be informative of the various pathways where zinc is a major functional factor in higher brain function.

Thus, here, we will summarize the behavioral phenotypes including effects on learning and memory abilities observed in various animal models of zinc deficiency, among them environmentally produced zinc deficient rodents and monkeys, as well as transgenic mouse models with targeted disruption of a particular zinc homeostasis gene. Moreover, we will shortly review the limited data obtained from human studies in comparison to the findings in zinc deficient animals.

## Behavioral phenotypes of environmentally induced zinc deficiency

Already in 1933, it was reported that zinc is essential for the growth of rats (Todd et al., 1933). That this is also true for humans was established in the 1960s by several reports from Prasad et al. on patients from the Middle East (Prasad et al., 1961, 1963a,b). However, besides overt clinical features such as growth retardation, skin irritations and hypogonadism, apart from mental lethargy, little has been reported on behavioral abnormalities in these patients. It was only in the 1990s, when a few studies in school-age children directly addressed the effects of low zinc diet on behavior (Gibson et al., 1989; Cavan et al., 1993a,b). Still today, the majority of data regarding behavioral alterations due to zinc deficiency comes from findings in animal models. Important studies are summarized in Table 1.

**Table 1:** **Summary of behavioral studies on zinc deficient animals**.

Phenotype	Test	Species	Age/time-point of deprivation	Reference
Impairment in learning and memory	Tolman-Honzik maze at PD44	Rat	PD1—PD21	Lokken et al. (1973)
	passive avoidance task (foot shock; shuttle box) at PD60	Rat	E15—E20	Halas and Sandstead (1975)
	avoidance task (foot shock; Skinner box) at PD11,14,17,20 and PD56,59,62,65	Rat	PD1—PD21	Halas et al. (1979)
	passive avoidance task (foot shock; shuttle box) at PD70	Mouse	E16—PD15	Golub et al. (1983)
	17-arm radial maze at PD100	Rat	PD1—PD18-21	Halas et al. (1983)
	visual discrimination task	Monkey	E111—PD116	Strobel and Sandstead (1984)
	17-arm radial maze at PD100	Rat	E1—PD23	Halas et al. (1986)
	Morris water maze	Rat	adult rat, acute hippoc. DDC and CaEDTA infusion	Frederickson et al. (1990)
	passive avoidance task (foot shock; shuttle box) at PD84	Rat	PD56—PD84	Takeda et al. (2000)
	Morris water maze at PD75	Rat	PD54—PD75	Keller et al. (2000)
	Morris water maze	Mouse	PD120 acute hippoc. DDC and CaEDTA infusion	Lassalle et al. (2000)
	fear conditioning (foot shock)	Mouse	PD91 acute hippoc. DEDTC and CaEDTA infusion	Daumas et al. (2004)
	Morris water maze at PD56	Rat	E1—PD21	Tahmasebi Boroujeni et al. (2009)
	fear conditioning and extinction (foot shock) at PD105	Mouse	PD84—PD105	Whittle et al. (2010)
	Morris water maze at PD56	Mouse	PD21—PD56	Gao et al. (2011)
	Morris water maze at PD56	Rat	E1—PD21	Yu et al. (2013)
Impairment in social behavior including increased emotionality (aggression/ anxiety)	interindividual aggression after shock at PD75	Rat	E14—E20	Halas et al. (1975)
	active avoidance (foot shock; shuttle box) at PD75	Rat	E14—E20	Sandstead et al. (1977)
	Taylor’s two-choice T-maze at PD49	Rat	E14—E20	Peters (1978)
	Open field test; elevated plus maze at PD35/42	Rat	PD28—PD35/42	Takeda et al. (2007)
	Open field test at PD56	Rat	PD28—PD56	Takeda et al. (2008)
	Open field test; light-dark box; elevated plus maze at PD77	Mouse	PD56—PD77	Whittle et al. (2009)
	maternal resident intruder test (aggression); maternal behavior test (social behavior) at PD105	Mouse	E1—E21	Grabrucker et al. (2014)
Enhanced stress response	Tolman-Honzik maze at PD44	Rat	PD1—PD21	Lokken et al. (1973)
	passive avoidance task (foot shock; shuttle box) at PD60	Rat	E15—E20	Halas and Sandstead (1975)
Depression—like behavior	light-dark box; Porsolt swim test at PD84	Rat	PD63—PD84	Tassabehji et al. (2008)
	forced swim test at PD42	Rat	PD28—PD42	Tamano et al. (2009)
	novelty suppressed feeding; tail suspension test; forced swim test; at PD77	Mouse	PD56—PD77	Whittle et al. (2009)
	forced swim test at PD42	Rat	PD28—PD42	Watanabe et al. (2010)
	forced swim test at PD35	Mouse	PD21—PD35	Młyniec et al. (2012)
	tail suspension test at PD35-91	Mouse	PD21—PD35-91	Młyniec and Nowak (2012)
	forced swim test at PD63	Mouse	PD21—PD63	Młyniec et al. (2013)
Impaired vocalization	ultrasonic vocalization at PD105	Mouse	E1—E21	Grabrucker et al. (2014)

*The table summarizes the most commonly reported phenotypes of pre- and postnatal zinc deficient animals (E: embryonic day; PD: postnatal day). The time-point given for behavioral testing indicates the age of the animals at the starting day of experiments*.

### Prenatal and perinatal zinc deficiency in animal models

Zinc deprivation is most frequently employed during the period of most rapid fetal brain growth during pregnancy and discontinued after birth. Behavioral testing is conducted later in adulthood (usually after sexual maturation). Thus, the tested animals do not suffer from acute zinc deficiency at the time of behavioral observation. Critical periods of susceptibility to zinc depletion during development have been identified in rodents (Halas, 1983). For example, severe zinc deprivation of pregnant rats during the first or all trimesters of pregnancy, leads to teratological effects. In contrast, mild zinc deprivation of pregnant rats during all trimesters or severe zinc deprivation only in the last third of gestation (embryonic day 14–20) does not induce malformations, but could still affect brain growth and function. Several studies on prenatal zinc deficient animals report characteristic behavioral effects, such as altered learning and memory, attention, and impaired social behavior. In general, the development of the normal associational network of neurons seems to be impaired under dietary zinc deprivation (Dreosti, 1983; Dvergsten et al., 1984).

#### Deficits in learning and memory

Mice with mild or moderate zinc deficiency from day 16 of gestation to postnatal day 15 were tested at 70 days of age in a passive avoidance task used to evaluate learning and memory. In this test, mice learn to avoid an environment in which an aversive stimulus (foot-shock) was previously delivered (Golub et al., 1983). Passive avoidance latencies were lower in both pre- and perinatal zinc deficient groups hinting towards impaired learning and memory. In another study, the effects of severe zinc deprivation during pregnancy and lactation were assessed in adult rats (at 100 days of age) using a radial maze, a task designed to investigate working memory, as well as short-term- and long-term memory. The results showed a severe learning deficit and some working memory deficit of prenatal zinc deficient animals when compared to controls (Halas et al., 1983, 1986). Further studies were able to repeat these findings. For example, it was reported that zinc deficiency during pregnancy and during lactation increased the time until rats learned to find a hidden platform when they were tested for spatial learning and memory in a Morris water maze at postnatal day 56 (Tahmasebi Boroujeni et al., 2009).

In another study, rats were similarly subjected to zinc deficiency during pregnancy and lactation. At 56 days of age, learning was tested using a Morris water maze. Again, prenatal zinc deficient animals needed more time finding the hidden platform and thus swam longer distances (Yu et al., 2013). Also offspring of rhesus monkeys with a severe zinc deficiency imposed during the third trimester of pregnancy display an impairment of learning later in life indicated by a difficulty in retaining previously learned visual discrimination tasks and by a difficulty in learning new tasks (Strobel and Sandstead, 1984; Golub et al., 1985).

Taken together, prenatal zinc deficiency may lead to lasting changes in synapse function or neuronal connectivity that manifest in reduced ability of learning and memory, dependent on the task, later in life.

#### Impaired social behavior (including enhanced stress response and increased emotionality)

Altered social behavior of prenatal zinc deficient mice was reported in multiple studies. Already in initial studies, zinc deprived males displayed a reduced affiliation in encounters with controls (Peters, 1978), which was attributed to increased aggressiveness. However, additional impairments in social behavior were reported in the offspring of mice that experienced moderate zinc deficiency during pregnancy. For example, prenatal zinc deficient mice display less maternal behavior towards their pups (Grabrucker et al., 2014).

Additionally, an enhanced stress response and emotionality after prenatal zinc deprivation was reported that was seen to influence the behavior of the animals in various test paradigms. For example, early studies investigating learning in prenatal zinc deficient rats found altered performance in shuttlebox shock avoidance tests using a negative reinforcer (shock), and the Tolman Honzik maze using a positive reinforcer (food) (Lokken et al., 1973; Halas and Sandstead, 1975). However, given that a sudden impairment in shock-motivated learning tasks was observed after normal initial learning, the shuttlebox test results might be explained by an enhanced response to stress. This enhanced stress response was also suggested to be the underlying cause of enhanced shock-elicited aggression in female (Halas et al., 1975) and male offspring (Sandstead et al., 1977; Peters, 1978). Moreover, in social encounters between experimental animals and control or zinc deficient rats, the zinc deficient rats were the social partners most often avoided given their increased aggressiveness (Peters, 1978). Similarly, prenatal zinc deficient female mice showed increased aggression in a resident intruder test when they were adults (Grabrucker et al., 2014).

In addition to rodent studies, studies using offspring of rhesus monkeys with a severe zinc deficiency imposed during the third trimester of pregnancy reported reduced activity, exploration, and play (Sandstead et al., 1978).

### Postnatal zinc deficiency in animal models

When behavior is tested during a period of zinc deprivation in immature animals, altered emotionality, lethargy, and deficits in learning, attention, and memory are most prominent. However, in mature animals, different or additional alterations can occur as well as the persistence of the phenotype after restitution of physiological zinc levels affected. For example, impaired learning behavior has been reported in zinc deficient animal models such as rats and rhesus monkeys (Golub et al., 1995). However, the time-point of zinc deficiency might determine, whether the effects are reversible given that zinc deficiency during early development elicits irrecoverable impairment, while the impairment of learning and memory was rescued in young adult rats by feeding a normal diet (Takeda et al., 2000). It is thus necessary to carefully evaluate the time period of zinc deprivation, the severity of deficiency, the use of pair-fed animals or animals fed *ad libitum* as controls, and the gender of zinc deficient animals if comparisons between different studies are to be undertaken. Additionally, other consequences of zinc deficiency have to be taken into account such as the occurrence of anorexia or growth retardation. For example, zinc deficient monkeys who developed behavioral deficits continued to grow during a period of accelerated post weaning growth, whereas monkeys that showed arrested growth displayed normal behavior (Golub et al., 1985). However, despite sometimes slightly different outcomes using postnatal zinc deficient animals that are most likely due to the non-standardized protocols used to generate these animals, few behavioral alterations are consistently reported.

#### Depression-like behavior (including lethargy)

Zinc deficiency is able to induce depressive-like behavior in adult rodents (Młyniec et al., 2014). In multiple studies, zinc deficient animals showed increased immobility time in the forced swim test and tail suspension test, test paradigms to evaluate depression-like behavior (Tassabehji et al., 2008; Tamano et al., 2009; Whittle et al., 2009; Watanabe et al., 2010; Młyniec and Nowak, 2012; Młyniec et al., 2012, 2013). These results obtained from rodent studies indicate that zinc deficiency in humans might not only be a consequence of depression but may contribute to the development of depressive-like behavior in some cases. Furthermore, zinc deficiency lowered the efficacy of several antidepressants (Tassabehji et al., 2008; Whittle et al., 2009; Młyniec and Nowak, 2012; Młyniec et al., 2012). In line with these observations, various studies report an antidepressant-like effect of zinc in some models of depression (Kroczka et al., 2000, 2001; Nowak et al., 2003, 2005; Cieślik et al., 2007; Sowa-Kućma et al., 2008; Tassabehji et al., 2008).

#### Deficits in learning, attention, and memory

In early studies, rats that had been zinc deprived during the late nursing period were investigated regarding their ability to remember the association of two stimuli previously built through conditioned learning. To that end, a tone coupled with a shock was applied at 11, 14, 17 or 20 days of age. Forty-two days later when the offspring were adults, their memory was tested using a Skinner box. Rats that had been trained to press a bar discontinued this behavior after the tone was presented again in anticipation of the expected shock, indicating memory of the association. Animals that were severely zinc deprived postnatally, exhibited poorer memory as adults (Halas et al., 1979). These early findings were followed by a multitude of studies that reported behavioral impairments regarding learning and memory in animals fed with a zinc restricted diet for several weeks. In particular, impaired spatial memory and extinction learning was consistently described (Keller et al., 2000; Whittle et al., 2010; Gao et al., 2011). For example, after 3 weeks of dietary zinc restriction, adult rats demonstrated significantly longer (86.0%) retrieval escape latencies (time to relocate the hidden platform after an initial trial) compared to controls in a Morris water maze test (Keller et al., 2000). Similarly, mice fed with a zinc deficient diet for 5 weeks showed impairments in spatial learning in the Morris water maze (Gao et al., 2011).

Zinc deficiency due to zinc chelating agents has also been used to assess the role of zinc in memory. Multiple studies have found that intra-hippocampal infusion of zinc chelators such as diethyldithiocarbamate (DEDTC) or CaEDTA alter learning and memory in rodents. DEDTC for example was shown to impair performance of animals in a “delayed matching-to-place” version of the water maze task (Frederickson et al., 1990). DEDTC injection shortly before training suppressed the decrease in time needed to find a platform which is normally observed on a second run. Another study investigated the behavior of DEDTC injected animals (intra-hippocampal) in a standard version of the Morris water maze (Lassalle et al., 2000). The results revealed that treatment during training prevented the mice from learning the location of a fixed hidden platform. In these experimental animals, the memory impairment occurred transitory and correlated with the time course of zinc chelation. This indicates that no substantial damage to the hippocampus due to the injection of chelator was responsible for the observed behavior.

Additionally, injection of zinc chelators (both of DEDTC and CaEDTA) was found to impair contextual fear conditioning (Daumas et al., 2004). While acute zinc chelation blocked the consolidation of contextual memory, it did not show an effect on the ability to recall information, once the memory has been formed (Sindreu and Storm, 2011). Thus, taken together, zinc deficiency, most likely by depleting synaptic zinc, impairs the acquisition or consolidation of hippocampus-dependent memory. Interestingly, under normal conditions, an age-related decrease in synaptic zinc is observed in mouse hippocampus. This decrease correlates with late-onset deficits in spatial reference memory (Adlard et al., 2010).

#### Altered emotionality (including aggression and anxiety)

Zinc deficiency was reported to have an anxiogenic effect in mice, which might also be related to depression-like behavior (Whittle et al., 2009). The increased anxiety-like behavior was similarly observed in rats. For example, in one study, after 14 days of zinc-deprivation, the frequency of entering the center zone in the open field test was decreased. Additionally, in the elevated plus-maze, the time spent in the open arms was decreased (Takeda et al., 2007). Both, the center zone of the open field and the open arms of the maze are unprotected areas, which are less frequently visited by anxious rodents. Additionally, after 14 days of zinc deficiency, isolated zinc deprived mice exhibited more aggressive behavior in the resident-intruder test, compared to isolated control mice and showed an increased duration of aggressive behavior indicating that aggressive behavior elicited by social isolation is enhanced by zinc deficiency (Takeda et al., 2008).

### Behavioral phenotypes of genetic animal models with targeted disruption of zinc homeostasis genes

To act within the brain, zinc has to be taken up from dietary sources in the small intestine, transported via the blood circulatory system, actively cross the blood-brain-barrier and be taken up into neurons or glial cells. To that end, a great variety of zinc transporters are expressed in various tissues. There are two families of zinc transporters in humans, *ZnT* (*SLC30A*) with at least 10 members and Zrt- and Irt-like protein (*ZIP*) (*SLC39A*) transporters with 15 members. ZIPs mostly mediate the uptake of zinc and ZnTs the export (into organelles or out of cells) of zinc and both, *ZnT* and *ZIP* transporters exhibit unique tissue-specific expression (Roohani et al., 2013).

Within the brain, *ZnT3* probably is the most investigated zinc transporter given that it promotes the influx of zinc into synaptic vesicles of glutamatergic neurons (Palmiter et al., 1996; Wenzel et al., 1997; Linkous et al., 2008). Knockout of *ZnT3* leads to the absence of histochemically reactive zinc in the terminals of zincergic neurons (Cole et al., 1999). The role of released zinc due to neuronal activation is not fully understood but it is suggested that zinc has a modifying role in signal transmission (Pan et al., 2011) and synaptic plasticity. Zinc containing glutamatergic neurons are found in most parts of the cerebral cortices and limbic structures, predominantly in the hippocampus (Frederickson et al., 2000). These brain regions play important roles in memory, emotions, behavior, motivation, and in the processing of sensory information. Therefore, it is likely that a lack of *ZnT3* might lead to disturbances in these brain functions. However, in most of the performed behavioral experiments, *ZnT3* knockout mice behaved similar to wildtype littermates. Nevertheless, some characteristic alterations could be observed such as age-dependent deficits in learning and memory.

Although young *ZnT3* knockout mice need more time to find the new location of a platform in the Morris water maze tests indicating slight deficits in reversal spatial learning (Cole et al., 2001; Martel et al., 2011), in general, young *ZnT3* knockout mice have normal learning and memory functions (Cole et al., 2000, 2001; Adlard et al., 2010). In contrast, adult *ZnT3* knockout mice, show deficits in learning and memory at 6 months of age in the Morris water maze (Adlard et al., 2010). Further, while no impairment in working memory was observed in a water version of the radial arm maze, impairments in working memory were observed in a rewarded-alternation T-maze (Cole et al., 2001; Sindreu et al., 2011). Tests for avoiding and memorizing threatening situations, revealed a normal passive avoidance behavior of *ZnT3* knockout mice and normal fear memory (Cole et al., 2001; Martel et al., 2010). However, if a too complex protocol for conditioning was used, *ZnT3* knockout mice show deficits in fear memory (Martel et al., 2010). Additionally, deficits in contextual discrimination and contextual fear memory have been observed in association with a higher rate of extinction (Martel et al., 2010; Sindreu et al., 2011). Thus, taken together, *ZnT3* mice, especially in old age, display some deficits in learning and memory.

In contrast,* ZnT3* knockout mice showed no differences in anxiety compared to their wildtype littermates (Cole et al., 2000, 2001; Adlard et al., 2010; Martel et al., 2011) and have comparable performances in motor coordination assessed by rotarod, pole and cagetop tests, and swim speed in water maze trials. Further, the loss of pre-synaptic zinc does not affect auditory, olfactory or visual abilities (Cole et al., 2000, 2001; Adlard et al., 2010; Martel et al., 2011) and *ZnT3* knockout mice show normal nociception in tail-flick assays and hot plate tests and normal pain sensitivity to electrical foot shocks (Cole et al., 2001; Martel et al., 2011).

Although *ZnT3* knockout mice showed increased social interactions, they failed to discriminate between a familiar and an unfamiliar mouse (Martel et al., 2011). Therefore, the increase in social interaction might be due to deficits in social and object recognition memory (Martel et al., 2011). In contrast to systemically zinc deficient animals, no increase in aggressive behavior has been observed during these tests (Martel et al., 2011). Similarly, no decrease in motivation assessed by the swim speed in Morris water maze and no increase in depression-like behavior evaluated by Porsolt’s forced-swim test was seen in *ZnT3* knockout mice (Martel et al., 2010, 2011). However, although *ZnT3* knockout mice do not develop spontaneous seizures they are prone to kainic acid induced seizures (Cole et al., 2000).

Several mouse models with targeted deletion of other zinc transporters have been described in detail for their phenotypic features and zinc levels. The available genetically modified animal models are listed in Table S1.

A homozygous deletion of *ZnT1* or *ZIP4* respectively is early embryonic lethal (Andrews et al., 2004; Dufner-Beattie et al., 2007). Both transporters show a high expression in tissues essential for zinc uptake such as intestine and the embryonic yolk sac (Langmade et al., 2000; Dufner-Beattie et al., 2003) and thus knockout was shown to have drastic consequences. Mutations in the *ZnT2* and the *ZnT4* gene may lead to zinc deficient milk produced by nursing mothers (lethal milk *lm* mice), which can in turn cause a deficiency in the pups (Piletz and Ganschow, 1978; Erway and Grider, 1984; Huang and Gitschier, 1997; Chowanadisai et al., 2006; Lee et al., 2013).

Knockout of *ZnT5*, *ZnT7* or *ZnT8* has tissue-specific effects and can influence metabolic pathways. *ZnT5* and *ZnT7* are thought to import zinc into the Golgi apparatus (Kambe et al., 2002; Kirschke and Huang, 2003) and thus, knockout mice show defects of acquisition and distribution of zinc (Inoue et al., 2002; Huang et al., 2007, 2012). *ZnT5* knockout mice show poor growth, osteopenia, low body fat, muscle weakness and male-specific cardiac death (Inoue et al., 2002), while knockout of *ZnT7* and *ZnT8* can lead to abnormal glucose and insulin metabolism (Lemaire et al., 2009; Nicolson et al., 2009). In humans, polymorphisms in the *ZnT8* gene are linked to an increased susceptibility for type 2 diabetes (Sladek et al., 2007; Table S1).

In contrast to *ZnT* knockout animals, the consequences of targeted deletion of *ZIP* transporters needs further investigation. The subfamily II of *ZIPs* that includes *ZIP1-3* might have important functions during the adaption to zinc deficiency and possibly can serve as backup system in dietary zinc uptake (Kambe et al., 2004). Deletion of mouse *ZIP1, 2* or *3* causes no obvious phenotypic effects when dietary zinc is present in sufficient concentrations. However, if zinc is reduced, these knockout animal models show a higher susceptibility to develop malformations and signs of zinc deficiency (Dufner-Beattie et al., 2005, 2006; Peters et al., 2007). This also holds true for a double or even a triple deletion of this subfamily of zinc transporters (Dufner-Beattie et al., 2006; Kambe et al., 2008; Table S1).

Moreover, mouse models are available for *ZIP8*, *13* and *14*, all having defects in growth or development (Fukada et al., 2008; Hojyo et al., 2011; Gálvez-Peralta et al., 2012). Loss of function of *ZIP8* leads to defects in organ morphogenesis, and hematopoiesis (Gálvez-Peralta et al., 2012). Knockout of *ZIP13* results in abnormal connective tissue development (Fukada et al., 2008) and knockout of *ZIP14* in impaired gluconeogenesis. Additionally, deletion of ZIP14 severely affects systemic growth (Hojyo et al., 2011). With the exception of *ZnT3* and *ZnT4*, where *lm* mutant mice show a mild ataxic phenotype, to our knowledge, no behavioral effects of the genetic ablation of the zinc transporter or a resulting zinc deficiency have been investigated so far.

Besides the zinc transporting proteins of the ZIP and ZnTs family, other factors contribute to zinc homeostasis in the brain, including Metallothioneins (MTs). MTs are small proteins (4–14 kDa) with very high metal binding capacity. Due to a high Cys content, metals such as zinc, cadmium and copper can be arranged in metal-thiolate clusters. The metals are located in two separate domains, which can in total bind up to seven metal ions. Under physiological conditions, zinc is plentiful compared to other putative binding metals and often the sole metallic constituent of MTs. However, under exposure to toxic metals, cadmium, mercury, platinum, lead, and bismuth might also be associated with MTs. In mammals, four groups of MT have been identified (*MT-1, MT-2, MT-3, MT-4*). There are wide variations of expression in different tissues and across different developmental states. Additionally, levels of MTs react to dietary metal contents. Therefore, MTs can act as zinc buffering proteins, temporarily storing the metal, but also participate through targeted release of zinc in signaling pathways. While the *MT-2* gene encodes a single MT-2A protein, the *MT-1* gene encodes many isoforms (MT-1A, MT-1B, MT-1E, MT-1F, MT-1G, MT-1H and MT-1X). Both, *MT-1* and *MT-2* are expressed ubiquitously including the brain (Hidalgo et al., 2001). *MT-3* and possibly *MT-4* in turn are especially enriched in the CNS (Maret, 2000; Hidalgo et al., 2001). Besides the known functions in heavy metal detoxification, and metal buffering and transporting, MT-3 was reported to play an additional role in neuromodulatory events in the brain. This additional feature is also reflected in the behavioral phenotypes of animals with targeted deletion of MTs.

The functions of the isoforms 1–3 concerning learning, memory and behavior were investigated in several publications using mouse models with specific deletions. *MT-1* and *MT-2* were shown to play a role in the regulation of neurobehavioral activity as well as in spatial cognitive functions with zinc acting as a regulating factor (Levin et al., 2006; McAuliffe et al., 2008; Itoh et al., 2010). A double knockout of *MT-1/MT-2* in mice leads to a decreased spontaneous locomotor activity and even a higher anxiety in the unfamiliar environment of the open field. Intriguingly, in the Morris water maze as well as in the 8-arm radial maze, the deletion of MT-1 and 2 causes disturbance in spatial learning (Levin et al., 2006; McAuliffe et al., 2008; Itoh et al., 2010). This defect in memory acquisition can even be enhanced by zinc depletion during growth in the young adult animal (Itoh et al., 2010). Moreover, it was reported that *MT-1/MT-2* null mice show a greater susceptibility to the neurobehavioral effects caused by a prenatal mercury exposure (Yoshida et al., 2005).

Mice lacking *MT-3* show a reduction in brain zinc levels as a result of the absence of MT-3 bound zinc. In contrast to the *MT-1/MT-2* double knockout mice, young adult *MT-3* knockout animals show no deficits in spatial learning (Erickson et al., 1997), memory (Koumura et al., 2009) and spontaneous locomotor activity (Erickson et al., 1997; Koumura et al., 2009). Further, no differences in the ability to learn and remember simple associations could be observed between knockout and wildtype mice. Equal results were obtained for 2-year-old mice. In contrast, *MT-3* knockout mice are not only much more susceptible to kainic acid induced seizures but also show a higher mortality due to greater severity of seizures with a longer duration of motor convulsions and a shorter latency to seizure-onset compared to wildtype littermates. These observations indicate a potential role for *MT-3* in the regulation of zinc during neuronal stimulation (Erickson et al., 1997). However, *ZnT3/MT-3* double knockout mice did not show enhanced seizure sensitivities to kainic acid compared to *ZnT3* or *MT-3* knockout mice (Cole et al., 2000).

Moreover, *MT-3* knockout mice exhibit deficits in social interactions in a novel environment manifesting in a significant shorter mean duration per contact (Koumura et al., 2009). Acoustic startle response tests indicate that *MT-3* knockout mice seem to have deficits in the ability to filter unnecessary information as shown in a reduced prepulse inhibition (Koumura et al., 2009). *MT-3* mice have been suggested as suitable subjects to investigate psychological disorders (Koumura et al., 2009). Taken together, these findings suggest that MT-1 and 2, compared to MT-3 isoforms have quite different functions in the CNS and have distinct influence on behavior, memory and learning.

When the concentration of intracellular free zinc reaches a certain limit, MTF-1 (metal-responsive transcription factor-1) is activated and induces the expression of, among other target genes, MTs which then bind and buffer the zinc ions, and ZnT1 that exports surplus free zinc (Murakami and Hirano, 2008). *MTF-1* knockout mice unfortunately have an embryonic lethal phenotype due to liver degeneration (Günes et al., 1998) and to our knowledge no behavioral data of a brain specific conditional *MTF-1* knockout has been reported so far.

For a comparative overview of phenotypes associated with knockout of zinc homeostasis genes see supplementary Table S1.

### Behavioral phenotypes of human patients with zinc deficiency

In humans, similar to animal models, zinc intake seems to be linked to a healthy mental function. Zinc deficiency may be associated with deficits in activity, attention, altered behavioral and emotional responses and impaired motor development caused by abnormal cerebellar function (Black, 1998). Especially at young age in times of accelerated growth, humans might display increased susceptibility to zinc deficiency. Additionally, several studies have found sex differences, suggesting that boys are more prone to develop zinc deficiency than girls (Cavan et al., 1993b; Sazawal et al., 1996).

There is a high incidence of zinc deficiency in patients suffering from ADHD, autism, SCZ, and depression. Notwithstanding whether cause or consequence, the effects of zinc deficiency might be observable in the behavior of the people affected by these disorders.

In line with the increased depression-like behavior in zinc deficient rodents (Tassabehji et al., 2008; Whittle et al., 2009), sheep and goat show behavioral signs of zinc deficiency (Nelson et al., 1984) such as allotriophagia, also called wool eating disease, which includes symptoms like growth retardation, diarrhea, poor appetite, hair loss, and atypical eating behavior of the own wool (Ott et al., 1964; Nelson et al., 1984; Suliman et al., 1988; Alhaji and Musa, 2012). Allotriophagia is associated with low blood zinc levels and the symptoms can be induced experimentally by feeding zinc deficient diets (Ott et al., 1964; Nelson et al., 1984; Suliman et al., 1988; Alhaji and Musa, 2012). The observed behavioral abnormalities in those animals were regarded as signs of depression associated with hypozincemia by several authors (Nelson et al., 1984; Suliman et al., 1988; Alhaji and Musa, 2012) and were shown to be reversible upon zinc supplementation (Ott et al., 1964; Nelson et al., 1984; Suliman et al., 1988; Alhaji and Musa, 2012). The described symptoms also correlate to some extent with the clinical signs of zinc deficiency in humans, and it is reported that depressed patients are often zinc deficient (Maes et al., 1994; Nowak et al., 2005; Levenson, 2006; Sowa-Kućma et al., 2008).

In particular, the severity of zinc deficiency seems to correlate with the severity of depressive symptoms in patients (Maes et al., 1994; Wójcik et al., 2006; Amani et al., 2010). Increased anxiety is often reported as part of psychiatric disorders, such as depression, panic attacks and post-traumatic stress disorder. Patients suffering from anxiety disorder were found to have significantly reduced plasma zinc levels compared to controls (Russo, 2011; Islam et al., 2013). Anxiety symptoms were significantly reduced after zinc supplementation (Russo, 2011). Moreover, the plasma zinc concentrations were found inversely related to teacher ratings of anxiety in 3–5 years old boys in the Head Start program (Hubbs-Tait et al., 2007). Additionally, anxiety- and depression-related disorders are often characterized by excessive aggression (Neumann et al., 2010). Intriguingly, a study reported that assaultive behavior of young men might be associated with zinc deficiency (Walsh et al., 1997). However, zinc deficiency is often accompanied with a low general nutritional status so that the occurrence of aggression and violent behavior might not only be associated with low zinc levels but also with general malnutrition. Aggressive behavior was shown diminished by the supplementation of minerals and vitamins (Schoenthaler and Bier, 2000).

Many infants with autism are suffering from marginal to severe zinc deficiency. Recent findings suggest a relationship of infantile zinc deficiency with autism (Yasuda and Tsutsui, 2013). Zinc deficiency has been reported to occur with very high incidence rate (up to 50%) in young autistic children (Yasuda et al., 2011) and often occurs along with copper overload in this disorder. In line with this, the Cu/Zn ratio was reported to correlate with the severity of symptoms associated with autism (Faber et al., 2009; Russo et al., 2012; Li et al., 2014). Some of the core features of autism, such as impaired social behavior, language and communication problems, and a restricted or stereotyped pattern of activities have also been found in prenatal zinc deficient mice (Grabrucker et al., 2014) that also display a synaptic dysregulation of zinc binding proteins of the Shank family. Mutations and deletion of SHANK genes have been described in autistic patients (Guilmatre et al., 2014). Along with the core features, co-morbidities occur frequently in autistic patients such as seizures and anxiety disorders that have been associated with zinc deficiency before. Thus, although a link between autism and prenatal zinc deficiency has not been investigated so far in detail, it is an emerging field of interest and further research is needed to determine the role of zinc deficiency in the pathology of autism.

Only few studies were conducted in which the zinc status of human participants was directly manipulated. However, in these studies, induction of severe zinc deficiency led to a sequence of reproducible effects. Clinical symptoms of zinc depletion such as skin lesions, diarrhea, and sore throats appear very early (King, 2011). The participants became anorexic after 2–3 days and developed neurosensory impairments in the sense of taste and smell. Subsequently, subjects experienced lethargy and displayed symptoms of depression, including increased irritability, and poor anger management. Additionally, an impairment of short-term memory was seen. At the last stage of the experiment, the participants showed signs of cerebellar dysfunction. The participants were then supplemented with 50 mg zinc per day, which led to a quick reversal of the symptoms (Henkin et al., 1975). Taken together, these data mirror many of the behavioral changes observed in zinc deficient animals. However, most studies that manipulated the zinc status of their participants mainly focused on other symptoms than behavioral abnormalities like an altered immune system function. Therefore, data on behavioral changes due to zinc deficiency in humans is only limited and there is need for further investigations.

## Conclusions

Due to the existence of only a very limited number of valid biomarkers, the clinical diagnosis especially of marginal zinc deficiency in humans remains problematic (Roohani et al., 2013). Determination of zinc levels in blood plasma or serum is currently the most commonly used method to evaluate the zinc status of an individual although there are major limitations in validity and reliability for the identification of mild zinc deficiency in individuals given that serum zinc concentrations may fluctuate by as much as 20% during a day (Hambidge et al., 1989). Assessment of zinc levels in hair samples via inductively coupled plasma mass spectrometry (ICP-MS) might be a preferable method. However, still, zinc deficiency, especially mild or transient deficiencies are hard to detect in humans and zinc deficiency is commonly overlooked. Thus, the exact prevalence of mild zinc deficiency is currently not known due to the inconsistency of clinical symptoms (Willoughby and Bowen, 2014). Particularly, mild zinc deficiency might not cause typical skin lesions or anorexia. Impaired night vision, depressed wound healing or altered immune function may not be perceived or associated with zinc status by an individual. Also, signs associated with zinc deficiency in babies that have been documented in several case reports, such as excessive crying, irritability, and inconsolability (Sivasubramanian and Henkin, 1978; Aggett et al., 1980) cannot be used as indicators given the many possible underlying causes for these symptoms. Thus, whereas for type 1 nutrients, where a low tissue concentration causes an impairment of one or more specific functions, deficiency of zinc as a type 2 nutrient gives rise to rather nonspecific symptoms, such as reduced growth, skin lesions, or infection. The tissue concentration of a type 2 nutrient is fixed and varies little even in times of depletion. In contrast, a small vulnerable pool that must be sustained by continuous dietary supply is depleted quickly (Golden, 1989). However, because zinc is a type 2 nutrient, the response to zinc repletion occurs also very fast (Golden, 1989). Compared to the symptoms mentioned above, changes in behavior, such as increased anxiety, irritability and depression might even be better markers for zinc deficiency.

### Behavioral impairments associated with zinc deficiency—lessons from animal studies

**Figure 1:**
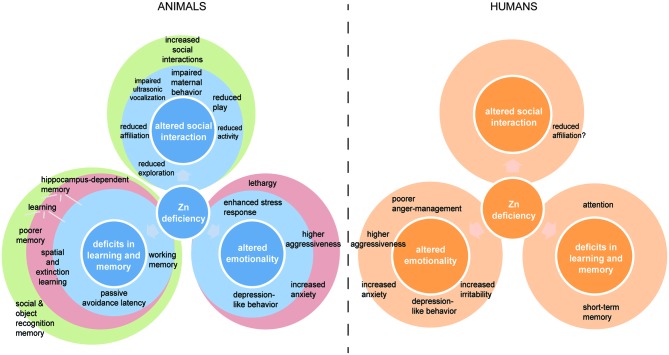
**Comparative overview of behavioral alterations reported associated with zinc deficiency in animals and humans**. Animal models for zinc deficiency (left) are distinguished into animals with pre- and perinatal zinc deprivation (blue color), postnatal zinc depletion (red color), and altered zinc homeostasis due to genetic manipulation (green color). Specific impairments are shown within a circle in case they only occurred in this particular animal model and within circles if they occurred in more than one group. Some of the behavioral alterations in animal models can be found mirrored in the behavior of human (right) zinc deficient individuals. Only impairments with strong validation are shown. Although possibly more similarities between zinc deficient humans and animals exist, more research on human zinc deficiency is necessary to exclude confounding factors.

Zinc deficiency in animals produces several characteristic and reproducible behavioral effects depending on the severity and developmental time-point of zinc deprivation (Figure 1). Altered emotionality including depression-like behavior, altered states of anxiety, and aggression, as well as altered social behavior and impaired memory and learning seem to be the most common outcomes of zinc deficiency in animal models. Behavioral effects of zinc deficiency are specific in animals with pre- and perinatal zinc deprivation, postnatal zinc depletion, and altered zinc homeostasis due to genetic manipulation, although some overlap exists. Some of the behavioral alterations in animal models can be found mirrored in the behavior of human zinc deficient individuals. However, the behavioral alterations observed in animal studies are produced by zinc deficiency in isolation from any other nutritional deprivation. The experimental animals are housed under optimal environmental conditions and do not suffer from secondary health problems. This is in contrast to the human situation, where zinc deficiency is mostly caused by malnutrition. Here, often multiple deficiencies of nutritional factors might exist in parallel and the physical condition in general might be affected. Thus, while in an animal study, a direct causal relationship between a behavioral alteration and zinc deficiency can be established, in human studies other factors might contribute to and modify the behavioral outcomes (Golub et al., 1995). Therefore, in human studies, it is necessary to not only correlate behavioral changes to the occurrence of zinc deficiency, but also to evaluate, how zinc supplementation might influence the observed phenotype. Here, clearly more controlled studies are needed in the future.

Animal studies reflect effects based on the timing and magnitude of zinc deficiency and thus provide information on the brain’s need for zinc at the time of the deficit. Severe fetal and neonatal zinc deficiency reveals an important role for zinc in cell division, neurogenesis, stem cell proliferation and growth hormone function, based on the observed teratogenesis and growth retardation. Mild fetal and neonatal zinc deficiency in turn seems to affect the behavior of the offspring, measurable even in adult life. Thus, zinc has an additional important role in synaptic plasticity establishing regional connectivity. Postnatal zinc deficiency also influences the behavior of animals. However, while social, and memory and learning impairments prevail in prenatal zinc deficient animals, acute postnatal zinc deficiency seems to lead to depression-like behavior, which might reflect a role for zinc in acute synapse function. Unfortunately, so far, no detailed behavioral studies of animal models for certain disorders such as autism, Alzheimer’s disease, Amyotrophic lateral sclerosis, or diabetes that have been subjected to zinc deficiency to observe a possible modulatory effect have been performed. Finally, genetic models underline the findings of studies with animals on a zinc deficient diet. Although very few data is available on the behavior of mice with targeted deletion of zinc transporters, mice with knockout of those zinc homeostasis genes that might play a major role in the brain, such as *ZnT3* and *MT-3* indeed show behavioral impairments. Unfortunately, mice with deletion of *ZnT1*, a post-synaptic zinc transporter, are embryonic lethal. However, it would not be surprising to find subtle behavioral phenotypes also in other *ZnT* and *ZIP* knockout mice, given that most of them are also expressed in the brain, or based on a developmental impairment that also affects CNS development. For example, *ZIP2* might be involved in neuronal zinc uptake and careful behavioral analysis might reveal subtle but interesting phenotypes. Finally, especially genetic deletion of brain zinc signaling genes such as *ZnT3*, *MT-3*, *ZIP1*, *ZIP3*, and the post-synaptic zinc receptor *GPR39*, reveals a role of zinc in influencing the susceptibility to develop seizures.

### Crosstalk between zinc deficiency and other disorders—lessons for human studies

**Figure 2:**
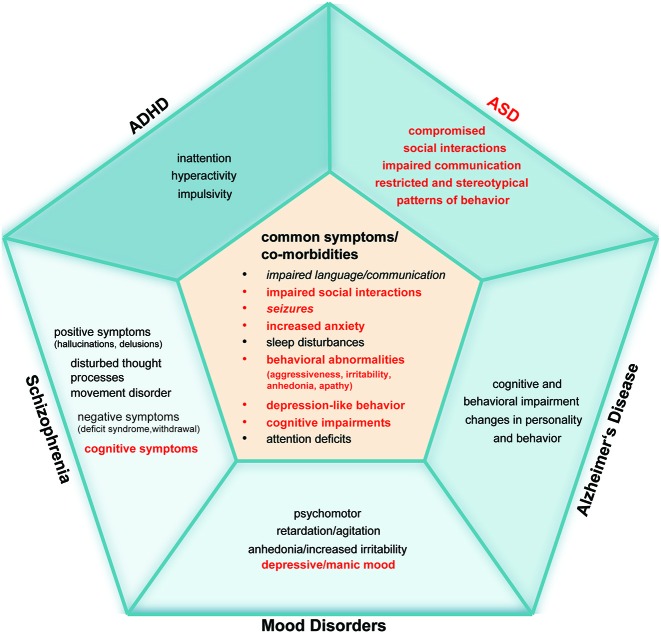
**Comparative overview of alterations reported in specific human diseases that share a behavioral pathology possibly associated with zinc deficiency**. Zinc deficiency is commonly reported in ASD, ADHD, Alzheimer’s Disease (AD), Schizophrenia (SCZ), and Mood Disorders (MD). Symptoms and comorbidities labeled in red are known to be associated with zinc deficiency. The inner pentagon summarizes symptoms and co-morbidities that co-occur in ASD, ADHD, AD, SCZ and MD (symptoms and co-morbidities that are written in italic do not appear in all five disorders: language and communication problems have not been reported to occur in MD. ADHD or MD patients do not suffer from seizures more frequently compared to healthy controls). “Impaired language and communication” includes impaired language functions, absence, delayed or reduced speech, speech problems and talking in a dull or monotonous way. Unstable relationships, difficult peer relations, the loss of empathy, social withdrawal and the reduced interest in social interactions are summarized by “impaired social interactions”. “Increased anxiety” implies panic disorder, posttraumatic stress disorder (PTSD), obsessive or compulsive disorder, and anxiety disorder. “Sleep disturbances” include trouble sleeping, daytime sleepiness such as longer “sleep onset latency,” frequent night-time awakenings and reduced sleep duration. Irritability, agitation, apathy, aggression, anhedonia, and self-injurious behavior are summarized by “behavioral abnormalities”. “Depression-like” behavior combines depressive mood, impaired motivation and initiative, loss of drive, decreased interest in previous activities, and feelings of hopelessness and helplessness. “Cognitive impairments” summarize thought disorder, lack of ability to begin or sustain planned activities, impaired executive functions (ability to understand information and use them to make decisions), problems with working memory, learning disability, impaired reasoning and handling of complex tasks, learning and memory deficits. “Attention deficits” implies inattention, trouble concentrating or making decisions and diminished concentration. The outer pentagons contain the core features of each disorder that are important for diagnosing the disorder. It is visible that the mentioned disorders share similarities, in particular in co-morbidities that frequently occur in these disorders, that are associated with zinc deficiency. Further research is needed to investigate, whether these co-morbidities are caused or modified by zinc deficiency.

The fact that mild zinc deficiency leads to rather unspecific symptoms in humans but occurs frequently as co-morbidity in several disorders makes the contribution of zinc deficiency to the pathology of a particular disorder hard to recognize. Therefore, even if diagnozed, zinc deficiency might not get the necessary attention in the treatment of a disorder although one could speculate about various ways how impaired zinc homeostasis might contribute mechanistically to the disease phenotype (Figure 2). In particular, the influence on synaptic function, inflammation and oxidative stress may be a common theme relevant to several disorders.

For example, zinc is a second messenger for immune cells and zinc deficiency affects T helper subset 1 cells and the activity and function of macrophages and their precursors, monocytes (Shankar and Prasad, 1998; Haase and Rink, 2007; Rosenkranz et al., 2011). An impaired immune system certainly is a modifying factor for any disorder, specifically also in neurodegenerative and neuropsychiatric disorders such as depression, but immune system alterations during pregnancy have also been reported as risk factor for autism (Grabrucker, 2012). Intriguingly, zinc deficiency also leads to a decrease in insulin-like growth factor 1 (IGF-1) levels independent from a reduction of total energy intake in rodents and humans (Cossack, 1991; Ninh et al., 1995; Ohlsson et al., 1998). Besides the consequences on the regulation of cell cycle and cell division that might underlie the observed growth retardation, IGF-1 was shown to be a potent factor in synaptic development and function, including effects on neurogenesis and synaptogenesis (O’Kusky et al., 2000). Indeed, IGF-1 levels were found significantly decreased in patients with autism compared to healthy controls (Riikonen et al., 2006) and IGF-1 treatment has shown beneficial effects in animal studies and was introduced as an experimental treatment for ASD (Canitano, 2014). Additionally, prenatal zinc deficiency in animal models influences *Shank3*, a well described autism-associated gene, at synapses (Grabrucker et al., 2014). It is thus possible that zinc deficiency, in particular prenatal and early postnatal zinc deficiency has a so far underestimated role in the etiology of autism. In line with this, zinc intake was positively related to social behavior in girls in a cohort of Egyptian children aged 7–10 years (Wachs et al., 1995).

Via the NF-κB pathway, zinc is able to down-regulate the production of inflammatory cytokines and thus might act as anti-inflammatory agent (Prasad et al., 2011). Oxidative stress and increased inflammatory cytokines have been shown to contribute to several diseases, especially those associated with aging. Especially elderly subjects are at risk to develop zinc deficiency, also in the Western world (Prasad et al., 1993, 2007; Briefel et al., 2000). A study conducted in five European countries reports an incidence rate of zinc deficiency in 31% of people over 60 years of age (Marcellini et al., 2006) with some variation between different countries. However, particularly hospitalized elderly individuals might be at risk to develop zinc deficiency, indicated by a study that reports a prevalence of 28% among this group (Pepersack et al., 2001). Thus, disorders occurring mostly in an aged population such as Alzheimer’s disease and other neurodegenerative disorders but also diabetes mellitus type 2 and atherosclerosis may be particularly influenced by zinc deficiency.

Alzheimer’s disease patients as well as patients suffering from dementia in general show an increased incidence rate of depression. Approximately 20–30% of patients with Alzheimer’s disease have depression. This rate seems to remain constant across dementia stages (Enache et al., 2011). Apart from depression, patients with dementia often present additional psychiatric symptoms and behavioral disturbances. Unfortunately, to our knowledge, no studies comparing the rate of zinc deficiency in this subgroup of demented patients with demented patients without co-morbidities have been performed so far. However, one study compared the prevalence of zinc deficiency in several psychogeriatric patient groups to the prevalence in a healthy control group and found an incidence rate of 41.0% in the patient group and 14.4% in the control group (Grønli et al., 2013). There were no significant differences between patients with depression as their main diagnosis and patients with depression as a co-morbid diagnosis, which might imply that in both groups, zinc deficiency might contribute to the occurrence of depression. Additionally, among the patients with dementia, a similarly high rate of zinc deficiency (48.5%) was found. The individuals with dementia in this patient group showed severe behavioral disturbances or psychiatric symptoms, such as psychosis, depression, or anxiety (Grønli et al., 2013).

The existence of diabetes mellitus is associated with anxiety disorders and doubles the probability of depression occurrence (Gavard et al., 1993; Ali et al., 2006; Knol et al., 2006; Golden et al., 2008), which was associated with hyperglycemia. However, zinc deficiency is also rather common in this patient group (Kinlaw et al., 1983). Finally, an association of depression and atherosclerosis has been reported in multiple studies (Saleptsis et al., 2011). It is thus possible that zinc deficiency is a modifier in several disorders mostly affecting the elderly and its outcome might be best visible by increasing anxiety and depression-like symptoms. On a molecular level, zinc deficiency might induce depression by a number of possible mechanism such as modification of neurotransmitter systems, particularly serotonergic and glutamatergic, neurotrophic factors, antioxidant mechanisms, and regulation of neuronal precursor cells (Cope and Levenson, 2010). For example, zinc levels influence the density of 5-HT_1A_ and 5-HT_2A_ serotonin receptors, modulate glutamate signaling via *N*-methyl-D-aspartate (NMDA) receptors, and alter the level of reactive oxygen species (ROS) and brain-derived neurotrophic factor (BDNF) and its tyrosine kinase receptor B (TrkB; Cope and Levenson, 2010).

However, the role of zinc in the human body is so manifold that the attempt to attribute a certain zinc dependent mechanism or pathway to the etiology or pathology of a disorder is doomed by the innumerable crosstalk between zinc dependent mechanisms that all might be affected in parallel but to a different extent. Moreover, to draw conclusions from affected zinc dependent functions on a certain observed behavioral outcome is difficult, especially given the magnitude of other cultural, social and physiological factors influencing human behavior. Nevertheless, based on the current knowledge from animal studies, it is safe to conclude that zinc deficiency influences animal behavior and will act on human behavior dependent on the developmental time-point of zinc deprivation and might modify the pathology of various diseases (Figure 2). It is visible that several pathologies such as ASD, ADHD, Alzheimer’s Disease, SCZ, and Mood Disorders share similarities, in particular in comorbidities that frequently occur in these disorders, that are associated with zinc deficiency. To investigate, whether these co-morbidities are caused or modified by zinc deficiency, detailed clinical studies have to be performed. In addition, in future, bioinformatics approaches will hopefully help to simulate the various possible outcomes of zinc deficiency on a molecular level and help to unravel the impact of altered zinc signaling in specific disorders.

## Conflict of interest statement

The authors declare that the research was conducted in the absence of any commercial or financial relationships that could be construed as a potential conflict of interest.

## References

[B1] Adlard P. A. Parncutt J. M. Finkelstein D. I. Bush A. I. (2010). Cognitive loss in zinc transporter-3 knock-out mice: a phenocopy for the synaptic and memory deficits of Alzheimer’s disease? J. Neurosci. 30, 1631–1636. 10.1523/jneurosci.5255-09.2010 20130173 PMC6633978

[B2] Aggett P. J. Atherton D. J. More J. Davey J. Delves H. T. Harries J. T. (1980). Symptomatic zinc deficiency in a breast-fed preterm infant. Arch. Dis. Child. 55, 547–550. 10.1136/adc.55.7.547 7192074 PMC1626780

[B3] Alhaji N. B. Musa I. G. (2012). Zinc deficiency (hypozincemia) in a lamb: clinical field case. IJAVMS 6, 349–352 10.5455/ijavms.20111106103000

[B4] Ali S. Stone M. A. Peters J. L. Davies M. J. Khunti K. (2006). The prevalence of co-morbid depression in adults with Type 2 diabetes: a systematic review and meta-analysis. Diabet. Med. 23, 1165–1173. 10.1111/j.1464-5491.2006.01943.x 17054590

[B5] Amani R. Saeidi S. Nazari Z. Nematpour S. (2010). Correlation between dietary zinc intakes and its serum levels with depression scales in young female students. Biol. Trace Elem. Res. 137, 150–158. 10.1007/s12011-009-8572-x 20013161

[B6] Andrews G. K. Wang H. Dey S. K. Palmiter R. D. (2004). Mouse zinc transporter 1 gene provides an essential function during early embryonic development. Genesis 40, 74–81. 10.1002/gene.20067 15452870

[B7] Bitanihirwe B. K. Cunningham M. G. (2009). Zinc: the brain’s dark horse. Synapse 63, 1029–1049. 10.1002/syn.20683 19623531

[B8] Black M. M. (1998). Zinc deficiency and child development. Am. J. Clin. Nutr. 68, 464–469. 9701161 10.1093/ajcn/68.2.464S PMC3137936

[B9] Briefel R. R. Bialostosky K. Kennedy-Stephenson J. McDowell M. A. Ervin R. B. Wright J. D. (2000). Zinc intake of US population findings from the third national health and nutrition survey 1988–1994. J. Nutr. 130, 1367–1373. 10.1093/jn/130.5.1367S 10801945

[B10] Canitano R. (2014). New experimental treatments for core social domain in autism spectrum disorders. Front. Pediatr. 2:61. 10.3389/fped.2014.00061 24999471 PMC4064155

[B12] Cavan K. R. Gibson R. S. Grazioso C. F. Isalgue A. M. Ruz M. Solomons N. W. (1993a). Growth and body composition of periurban Guatemalan children in relation to zinc status: a longitudinal zinc intervention trial. Am. J. Clin. Nutr. 57, 344–352. 8438768 10.1093/ajcn/57.3.344

[B11] Cavan K. R. Gibson R. S. Grazioso C. F. Isalgue A. M. Ruz M. Solomons N. W. (1993b). Growth and body composition of periurban Guatemalan children in relation to zinc status: a cross-sectional study. Am. J. Clin. Nutr. 57, 334–343. 8438767 10.1093/ajcn/57.3.334

[B13] Chowanadisai W. Lönnerdal B. Kelleher S. L. (2006). Identification of a mutation in SLC30A2 (ZnT-2) in women with low milk zinc concentration that results in transient neonatal zinc deficiency. J. Biol. Chem. 281, 39699–39707. 10.1074/jbc.m605821200 17065149

[B14] Cieślik K. Klenk-Majewska B. Danilczuk Z. Wróbel A. Łupina T. Ossowska G. (2007). Influence of zinc supplementation on imipramine effect in a chronic unpredictable stress (CUS) model in rats. Pharmacol. Rep. 59, 46–52. 17377205

[B15] Cole T. B. Martyanova A. Palmiter R. D. (2001). Removing zinc from synaptic vesicles does not impair spatial learning, memory, or sensorimotor functions in the mouse. Brain Res. 891, 253–265. 10.1016/s0006-8993(00)03220-0 11164830

[B16] Cole T. B. Robbins C. A. Wenzel H. J. Schwartzkroin P. A. Palmiter R. D. (2000). Seizures and neuronal damage in mice lacking vesicular zinc. Epilepsy Res. 39, 153–169. 10.1016/s0920-1211(99)00121-7 10759303

[B17] Cole T. B. Wenzel H. J. Kafer K. E. Schwartzkroin P. A. Palmiter R. D. (1999). Elimination of zinc from synaptic vesicles in the intact mouse brain by disruption of the ZnT3 gene. Proc. Natl. Acad. Sci. U S A 96, 1716–1721. 10.1073/pnas.96.4.1716 9990090 PMC15571

[B18] Cope E. C. Levenson C. W. (2010). Role of zinc in the development and treatment of mood disorders. Curr. Opin. Clin. Nutr. Metab. Care 13, 685–689. 10.1097/mco.0b013e32833df61a 20689416

[B19] Cossack Z. T. (1991). Decline in somatomedin-C, insulin-like growth factor-1, with experimentally induced zinc deficiency in human subjects. Clin. Nutr. 10, 284–291. 10.1016/0261-5614(91)90008-z 16839933

[B20] Daumas S. Halley H. Lassalle J. M. (2004). Disruption of hippocampal CA3 network: effects on episodic-like memory processing in C57BL/6J mice. Eur. J. Neurosci. 20, 597–600. 10.1111/j.1460-9568.2004.03484.x 15233771

[B21] Dreosti I. E. (1983). “Zinc and the central nervous system,” in Neurobiology of the Trace Elements (Vol. 1), eds Smith I. E. Dreosti R. M. (Clifton: Humana), 135–162.

[B22] Dufner-Beattie J. Huang Z. L. Geiser J. Xu W. Andrews G. K. (2005). Generation and characterization of mice lacking the zinc uptake transporter ZIP3. Mol. Cell. Biol. 25, 5607–5615. 10.1128/mcb.25.13.5607-5615.2005 15964816 PMC1156975

[B23] Dufner-Beattie J. Huang Z. L. Geiser J. Xu W. Andrews G. K. (2006). Mouse ZIP1 and ZIP3 genes together are essential for adaptation to dietary zinc deficiency during pregnancy. Genesis 44, 239–251. 10.1002/dvg.20211 16652366

[B24] Dufner-Beattie J. Wang F. Kuo Y. M. Gitschier J. Eide D. Andrews G. K. (2003). The acrodermatitis enteropathica gene ZIP4 encodes a tissue-specific, zinc-regulated zinc transporter in mice. J. Biol. Chem. 278, 33474–33481. 10.1074/jbc.m305000200 12801924

[B25] Dufner-Beattie J. Weaver B. P. Geiser J. Bilgen M. Larson M. Xu W.. (2007). The mouse acrodermatitis enteropathica gene Slc39a4 (Zip4) is essential for early development and heterozygosity causes hypersensitivity to zinc deficiency. Hum. Mol. Genet. 16, 1391–1399. 10.1093/hmg/ddm088 17483098

[B26] Dvergsten C. L. Johnson L. A. Sandstead H. H. (1984). Alterations in the postnatal development of the cerebellar cortex due to zinc deficiency. III. Impaired dendritic differentiation of basket and stellate cells. Brain Res. 318, 21–26. 10.1016/0165-3806(84)90058-0 6488052

[B27] Enache D. Winblad B. Aarsland D. (2011). Depression in dementia: epidemiology, mechanisms and treatment. Curr. Opin. Psychiatry 24, 461–472. 10.1097/yco.0b013e32834bb9d4 21926624

[B28] Erickson J. C. Hollopeter G. Thomas S. A. Froelick G. J. Palmiter R. D. (1997). Disruption of the metallothionein-III gene in mice: analysis of brain zinc, behavior and neuron vulnerability to metals, aging and seizures. J. Neurosci. 17, 1271–1281. 9006971 10.1523/JNEUROSCI.17-04-01271.1997 PMC6793742

[B29] Erway L. C. Grider A. Jr. (1984). Zinc metabolism in lethal-milk mice. Otolith, lactation and aging effects. J. Hered. 75, 480–484. 6512240 10.1093/oxfordjournals.jhered.a109990

[B30] Evans S. A. Overton J. M. Alshingiti A. Levenson C. W. (2004). Regulation of metabolic rate and substrate utilization by zinc deficiency. Metabolism 53, 727–732. 10.1016/s0026-0495(04)00079-4 15164319

[B31] Faber S. Zinn G. M. Kern J. C. Kingston H. M. (2009). The plasma zinc/serum copper ratio as a biomarker in children with autism spectrum disorders. Biomarkers 14, 171–180. 10.1080/13547500902783747 19280374

[B32] Fischer Walker C. L. Ezzati M. Black R. E. (2009). Global and regional child mortality and burden of disease attributable to zinc deficiency. Eur. J. Clin. Nutr. 63, 591–597. 10.1038/ejcn.2008.9 18270521

[B34] Frederickson R. E. Frederickson C. J. Danscher G. (1990). In situ binding of bouton zinc reversibly disrupts performance on a spatial memory task. Behav. Brain Res. 38, 25–33. 10.1016/0166-4328(90)90021-6 2161241

[B33] Frederickson C. J. Suh S. W. Silva D. Frederickson C. J. Thompson R. B. (2000). Importance of zinc in the central nervous system: the zinc-containing neuron. J. Nutr. 130, 1471–1483. 10801962 10.1093/jn/130.5.1471S

[B35] Fukada T. Civic N. Furuichi T. Shimoda S. Mishima K. Higashiyama H.. (2008). The zinc transporter SLC39A13/ZIP13 is required for connective tissue development; its involvement in BMP/TGF-beta signaling pathways. PLoS One. 3:e3642. 10.1371/journal.pone.0003642 18985159 PMC2575416

[B36] Gálvez-Peralta M. He L. Jorge-Nebert L. F. Wang B. Miller M. L. Eppert B. L.. (2012). ZIP8 zinc transporter: indispensable role for both multiple-organ organogenesis and hematopoiesis in utero. PLoS One. 7:e36055. 10.1371/journal.pone.0036055 22563477 PMC3341399

[B37] Gao H. L. Xu H. Xin N. Zheng W. Chi Z. H. Wang Z. Y. (2011). Disruption of the CaMKII/CREB signaling is associated with zinc deficiency-induced learning and memory impairments. Neurotox. Res. 19, 584–591. 10.1007/s12640-010-9206-y 20593259

[B38] Gavard J. A. Lustman P. J. Clouse R. E. (1993). Prevalence of depression in adults with diabetes. An epidemiological evaluation. Diabetes Care 16, 1167–1178. 10.2337/diacare.16.8.1167 8375247

[B39] Gibson R. S. Vanderkooy P. D. MacDonald A. C. Goldman A. Ryan B. A. Berry M. (1989). A growth-limiting, mild zinc-deficiency syndrome in some southern Ontario boys with low height percentiles. Am. J. Clin. Nutr. 49, 1266–1273. 2729165 10.1093/ajcn/49.6.1266

[B40] Golden M. H. N. (1989). “The diagnosis of zinc deficiency,” in Zinc in Human Biology, ed Mills C. F. (London, UK: Springer-Verlag), 173–181.

[B41] Golden S. H. Lazo M. Carnethon M. Bertoni A. G. Schreiner P. J. Diez Roux A. V.. (2008). Examining a bidirectional association between depressive symptoms and diabetes. JAMA 299, 2751–2759. 10.1001/jama.299.23.2751 18560002 PMC2648841

[B42] Golub M. S. Gershwin M. E. Hurley L. S. Hendrickx A. G. Saito W. Y. (1985). Studies of marginal zinc deprivation in rhesus monkeys: infant behavior. Am. J. Clin. Nutr. 42, 1229–1239. 4072958 10.1093/ajcn/42.6.1229

[B43] Golub M. S. Gershwin M. E. Vijayan V. K. (1983). Passive avoidance performance of mice fed marginally or severely zinc deficient diets during post-embryonic brain development. Physiol. Behav. 30, 409–413. 10.1016/0031-9384(83)90145-2 6867137

[B44] Golub M. S. Keen C. L. Gershwin M. E. Hendrickx A. G. (1995). Developmental zinc deficiency and behavior. J. Nutr. 125, 2263–2271. 7623165 10.1093/jn/125.suppl_8.2263S

[B45] Grabrucker A. M. (2012). Environmental factors in autism. Front. Psychiatry 3:118. 10.3389/fpsyt.2012.00118 23346059 PMC3548163

[B46] Grabrucker A. M. (2014). A role for synaptic zinc in ProSAP/Shank PSD scaffold malformation in autism spectrum disorders. Dev. Neurobiol. 74, 136–146. 10.1002/dneu.22089 23650259 PMC4272576

[B48] Grabrucker S. Jannetti L. Eckert M. Gaub S. Chhabra R. Pfaender S.. (2014). Zinc deficiency dysregulates the synaptic ProSAP/Shank scaffold and might contribute to autism spectrum disorders. Brain 137, 137–152. 10.1093/brain/awt303 24277719

[B47] Grabrucker A. M. Knight M. J. Proepper C. Bockmann J. Joubert M. Rowan M.. (2011). Concerted action of zinc and ProSAP/Shank in synaptogenesis and synapse maturation. EMBO J. 30, 569–581. 10.1038/emboj.2010.336 21217644 PMC3034012

[B49] Grønli O. Kvamme J. M. Friborg O. Wynn R. (2013). Zinc deficiency is common in several psychiatric disorders. PLoS One 8:e82793. 10.1371/journal.pone.0082793 24367556 PMC3868572

[B50] Guilmatre A. Huguet G. Delorme R. Bourgeron T. (2014). The emerging role of SHANK genes in neuropsychiatric disorders. Dev. Neurobiol. 74, 113–122. 10.1002/dneu.22128 24124131

[B51] Günes C. Heuchel R. Georgiev O. Müller K. H. Lichtlen P. Blüthmann H.. (1998). Embryonic lethality and liver degeneration in mice lacking the metal-responsive transcriptional activator MTF-1. EMBO J. 17, 2846–2854. 10.1093/emboj/17.10.2846 9582278 PMC1170625

[B52] Haase H. Rink L. (2007). Signal transduction in monocytes: the role of zinc ions. Biometals 20, 579–585. 10.1007/s10534-006-9029-8 17453150

[B53] Halas E. S. (1983). “Behavioral changes accompanying zinc deficiency in animals,” in Neurobiology of the Trace Elements (Vol. 1), Trace Element Neurobiology and Deficiencies, eds Dreosti I. E. Smith R. M. (Clifton, NJ: Humana Press), 213–243.

[B54] Halas E. S. Eberhardt M. J. Diers M. A. Sandstead H. H. (1983). Learning and memory impairment in adult rats due to severe zinc deficiency during lactation. Physiol. Behav. 30, 371–381. 10.1016/0031-9384(83)90140-3 6867134

[B55] Halas E. S. Hanlon M. J. Sandstead H. H. (1975). Intrauterine nutrition and aggression. Nature 257, 221–222. 10.1038/257221a0 1172194

[B56] Halas E. S. Heinrich M. D. Sandstead H. H. (1979). Long term memory deficits in adult rats due to postnatal malnutrition. Physiol. Behav. 22, 991–997. 10.1016/0031-9384(79)90345-7 574290

[B57] Halas E. S. Hunt C. D. Eberhardt M. J. (1986). Learning and memory disabilities in young adult rats from mildly zinc deficient dams. Physiol. Behav. 37, 451–458. 10.1016/0031-9384(86)90205-2 3749304

[B58] Halas E. S. Sandstead H. H. (1975). Some effects of prenatal zinc deficiency on behavior of the adult rat. Pediatr. Res. 9, 94–97. 10.1203/00006450-197509020-00007 1118197

[B60] Hambidge M. (2000). Human zinc deficiency. J. Nutr. 130, 1344–1349. 10801941 10.1093/jn/130.5.1344S

[B59] Hambidge K. M. Goodall M. J. Stall C. Pritts J. (1989). Post-prandial and daily changes in plasma zinc. J. Trace Elem. Electrolytes Health Dis. 3, 55–57. 2535321

[B61] Henkin R. I. Patten B. M. Re P. K. Bronzert D. A. (1975). A syndrome of acute zinc loss. Cerebellar dysfunction, mental changes, anorexia and taste and smell dysfunction. Arch. Neurol. 32, 745–751. 10.1001/archneur.1975.00490530067006 1180744

[B62] Hidalgo J. Aschner M. Zatta P. Vasák M. (2001). Roles of the metallothionein family of proteins in the central nervous system. Brain Res. Bull. 55, 133–145. 10.1016/s0361-9230(01)00452-x 11470309

[B63] Hojyo S. Fukada T. Shimoda S. Ohashi W. Bin B. H. Koseki H.. (2011). The zinc transporter SLC39A14/ZIP14 controls G-protein coupled receptor-mediated signaling required for systemic growth. PLoS One. 6:e18059. 10.1371/journal.pone.0018059 21445361 PMC3062567

[B64] Huang L. Gitschier J. (1997). A novel gene involved in zinc transport is deficient in the lethal milk mouse. Nat. Genet. 17, 292–297. 10.1038/ng1197-292 9354792

[B65] Huang L. Kirschke C. P. Lay Y. A. Levy L. B. Lamirande D. E. Zhang P. H. (2012). Znt7-null mice are more susceptible to diet-induced glucose intolerance and insulin resistance. J. Biol. Chem. 287, 33883–33896. 10.1074/jbc.m111.309666 22854958 PMC3460483

[B66] Huang L. Yu Y. Y. Kirschke C. P. Gertz E. R. Lloyd K. K. (2007). Znt7 (Slc30a7)-deficient mice display reduced body zinc status and body fat accumulation. J. Biol. Chem. 282, 37053–37063. 10.1074/jbc.m706631200 17954933

[B67] Hubbs-Tait L. Kennedy T. S. Droke E. A. Belanger D. M. Parker J. R. (2007). Zinc, iron and lead: relations to head start children’s cognitive scores and teachers’ ratings of behavior. J. Am. Diet. Assoc. 107, 128–133. 10.1016/j.jada.2006.10.001 17197281

[B68] Inoue K. Matsuda K. Itoh M. Kawaguchi H. Tomoike H. Aoyagi T.. (2002). Osteopenia and male-specific sudden cardiac death in mice lacking a zinc transporter gene, Znt5. Hum. Mol. Genet. 11, 1775–1784. 10.1093/hmg/11.15.1775 12095919

[B69] Islam M. R. Ahmed M. U. Mitu S. A. Islam M. S. Rahman G. K. Qusar M. M.. (2013). Comparative analysis of serum zinc, copper, manganese, iron, calcium and magnesium level and complexity of interelement relations in generalized anxiety disorder patients. Biol. Trace Elem. Res. 154, 21–27. 10.1007/s12011-013-9723-7 23754591

[B70] Itoh T. Nakai K. Satoh M. Satoh C. Kameo S. Nakagi Y. (2010). Effect of Zinc deficiency on the behavior of Metallothionein-I, II knockout mice. Biomed. Res. Trace Elem. 21, 204–213 10.11299/brte.21.204

[B71] Jan H. H. Chen I. T. Tsai Y. Y. Chang Y. C. (2002). Structural role of zinc ions bound to postsynaptic densities. J. Neurochem. 83, 525–534. 10.1046/j.1471-4159.2002.01093.x 12390514

[B72] Kambe T. Geiser J. Lahner B. Salt D. E. Andrews G. K. (2008). Slc39a1 to 3 (subfamily II) Zip genes in mice have unique cell-specific functions during adaptation to zinc deficiency. Am. J. Physiol. Regul. Integr. Comp. Physiol. 294, R1474–R1481. 10.1152/ajpregu.00130.2008 18353881 PMC2376821

[B73] Kambe T. Narita H. Yamaguchi-Iwai Y. Hirose J. Amano T. Sugiura N.. (2002). Cloning and characterization of a novel mammalian zinc transporter, zinc transporter 5, abundantly expressed in pancreatic beta cells. J. Biol. Chem. 277, 19049–19055. 10.1074/jbc.m200910200 11904301

[B74] Kambe T. Yamaguchi-Iwai Y. Sasaki R. Nagao M. (2004). Overview of mammalian zinc transporters. Cell. Mol. Life Sci. 61, 49–68. 10.1007/s00018-003-3148-y 14704853 PMC11138893

[B75] Keen C. L. Lonnerdal B. Golub M. S. Olin K. L. Graham T. W. Uriu-Hare J. Y.. (1993). Effect of the severity of maternal zinc deficiency on pregnancy outcome and infant zinc status in rhesus monkeys. Pediatr. Res. 33, 233–241. 10.1203/00006450-199303000-00005 8460059

[B76] Keller K. A. Chu Y. Grider A. Coffield J. A. (2000). Supplementation with L-histidine during dietary zinc repletion improves short-term memory in zinc-restricted young adult male rats. J. Nutr. 130, 1633–1640. 10827222 10.1093/jn/130.6.1633

[B77] King J. C. (2011). Zinc: an essential but elusive nutrient. Am. J. Clin. Nutr. 94, 679–684. 10.3945/ajcn.110.005744 21715515 PMC3142737

[B78] Kinlaw W. B. Levine A. S. Morley J. E. Silvis S. E. McClain C. J. (1983). Abnormal zinc metabolism in type II diabetes mellitus. Am. J. Med. 75, 273–277. 10.1016/0002-9343(83)91205-6 6881179

[B79] Kirschke C. P. Huang L. (2003). ZnT7, a novel mammalian zinc transporter, accumulates zinc in the Golgi apparatus. J. Biol. Chem. 278, 4096–4102. 10.1074/jbc.m207644200 12446736

[B80] Knol M. J. Twisk J. W. Beekman A. T. Heine R. J. Snoek F. J. Pouwer F. (2006). Depression as a risk factor for the onset of type 2 diabetes mellitus. A meta-analysis. Diabetologia 49, 837–845. 10.1007/s00125-006-0159-x 16520921

[B81] Koumura A. Kakefuda K. Honda A. Ito Y. Tsuruma K. Shimazawa M.. (2009). Metallothionein-3 deficient mice exhibit abnormalities of psychological behaviors. Neurosci. Lett. 467, 11–14. 10.1016/j.neulet.2009.09.051 19799968

[B82] Kroczka B. Branski P. Palucha A. Pilc A. Nowak G. (2001). Antidepressant-like properties of zinc in rodent forced swim test. Brain Res. Bull. 55, 297–300. 10.1016/s0361-9230(01)00473-7 11470330

[B83] Kroczka B. Zieba A. Dudek D. Pilc A. Nowak G. (2000). Zinc exhibits an antidepressant-like effect in the forced swimming test in mice. Pol. J. Pharmacol. 52, 403–406. 11334234

[B84] Langmade S. J. Ravindra R. Daniels P. J. Andrews G. K. (2000). The transcription factor MTF-1 mediates metal regulation of the mouse ZnT1 gene. J. Biol. Chem. 275, 34803–34809. 10.1074/jbc.m007339200 10952993

[B85] Lassalle J. M. Bataille T. Halley H. (2000). Reversible inactivation of the hippocampal mossy fiber synapses in mice impairs spatial learning, but neither consolidation nor memory retrieval, in the Morris navigation task. Neurobiol. Learn. Mem. 73, 243–257. 10.1006/nlme.1999.3931 10775494

[B86] Lee S. Hennigar S. R. Alam S. Kelleher S. L. (2013). ZnT2-null mice have distinct morphological defects in the mammary gland that impair development and function. FASEB J. 27, 733.7.

[B87] Lemaire K. Ravier M. A. Schraenen A. Creemers J. W. Van de Plas R. Granvik M.. (2009). Insulin crystallization depends on zinc transporter ZnT8 expression, but is not required for normal glucose homeostasis in mice. Proc. Natl. Acad. Sci. U S A. 106, 14872–14877. 10.1073/pnas.0906587106 19706465 PMC2736467

[B88] Levenson C. W. (2006). Zinc: the new antidepressant? Nutr. Rev. 64, 39–42. 10.1111/j.1753-4887.2006.tb00171.x 16491668

[B89] Levin E. D. Perraut C. Pollard N. Freedman J. H. (2006). Metallothionein expression and neurocognitive function in mice. Physiol. Behav. 87, 513–518. 10.1016/j.physbeh.2005.11.014 16430929

[B90] Li S. O. Wang J. L. Bjørklund G. Zhao W. N. Yin C. H. (2014). Serum copper and zinc levels in individuals with autism spectrum disorders. Neuroreport 25, 1216–1220. 10.1097/wnr.0000000000000251 25162784

[B91] Linkous D. H. Flinn J. M. Koh J. Y. Lanzirotti A. Bertsch P. M. Jones B. F.. (2008). Evidence that the ZNT3 protein controls the total amount of elemental zinc in synaptic vesicles. J. Histochem. Cytochem. 56, 3–6. 10.1369/jhc.6a7035.2007 17712179 PMC2323120

[B92] Lokken P. M. Halas E. S. Sandstead H. H. (1973). Influence of zinc deficiency on behavior. Proc. Soc. Exp. Biol. Med. 144, 680–682. 10.3181/00379727-144-37661 4746943

[B93] Lu Y. M. Taverna F. A. Tu R. Ackerley C. A. Wang Y. T. Roder J. (2000). Endogenous Zn(2+) is required for the induction of long-term potentiation at rat hippocampal mossy fiber-CA3 synapses. Synapse 38, 187–197. 10.1002/1098-2396(200011)38:2<187::aid-syn10>3.0.co;2-r 11018793

[B94] Maes M. D’Haese P. C. Scharpé S. D’Hondt P. Cosyns P. De Broe M. E. (1994). Hypozincemia in depression. J. Affect. Disord. 31, 135–140. 10.1016/0165-0327(94)90117-1 8071476

[B95] Marcellini F. Giuli C. Papa R. Gagliardi C. Dedoussis G. Herbein G.. (2006). Zinc status, psychological and nutritional assessment in old people recruited in five European countries: Zincage study. Biogerontology 7, 339–345. 10.1007/s10522-006-9048-4 16969711

[B96] Maret W. (2000). The function of zinc metallothionein: a link between cellular zinc and redox state. J. Nutr. 130, 1455–1458. 10801959 10.1093/jn/130.5.1455S

[B97] Martel G. Hevi C. Friebely O. Baybutt T. Shumyatsky G. P. (2010). Zinc transporter 3 is involved in learned fear and extinction, but not in innate fear. Learn. Mem. 17, 582–590. 10.1101/lm.1962010 21036893 PMC2981414

[B98] Martel G. Hevi C. Kane-Goldsmith N. Shumyatsky G. P. (2011). Zinc transporter ZnT3 is involved in memory dependent on the hippocampus and perirhinal cortex. Behav. Brain Res. 223, 233–238. 10.1016/j.bbr.2011.04.020 21545813 PMC3111813

[B99] McAuliffe J. J. Joseph B. Hughes E. Miles L. Vorhees C. V. (2008). Metallothionein I,II deficient mice do not exhibit significantly worse long-term behavioral outcomes following neonatal hypoxia-ischemia: MT-I,II deficient mice have inherent behavioral impairments. Brain Res. 1190, 175–185. 10.1016/j.brainres.2007.11.038 18083145

[B100] Młyniec K. Budziszewska B. Reczyński W. Doboszewska U. Pilc A. Nowak G. (2013). Zinc deficiency alters responsiveness to antidepressant drugs in mice. Pharmacol. Rep. 65, 579–592. 10.1016/s1734-1140(13)71035-1 23950580

[B101] Młyniec K. Davies C. L. Budziszewska B. Opoka W. Reczyński W. Sowa-Kućma M.. (2012). Time course of zinc deprivation-induced alterations of mice behavior in the forced swim test. Pharmacol. Rep. 64, 567–575. 10.1016/s1734-1140(12)70852-6 22814010

[B102] Młyniec K. Davies C. L. de Agüero Sánchez I. G. Pytka K. Budziszewska B. Nowak G. (2014). Essential elements in depression and anxiety. Part I. Pharmacol. Rep. 66, 534–544. 10.1016/j.pharep.2014.03.001 24948052

[B103] Młyniec K. Nowak G. (2012). Zinc deficiency induces behavioral alterations in the tail suspension test in mice. Effect of antidepressants. Pharmacol. Rep. 64, 249–255. 10.1016/s1734-1140(12)70762-4 22661173

[B104] Murakami M. Hirano T. (2008). Intracellular zinc homeostasis and zinc signaling. Cancer Sci. 99, 1515–1522. 10.1111/j.1349-7006.2008.00854.x 18754861 PMC11158020

[B105] Nelson D. R. Wolff W. A. Blodgett D. J. Luecke B. Ely R. W. Zachary J. F. (1984). Zinc deficiency in sheep and goats: three field cases. J. Am. Vet. Med. Assoc. 184, 1480–1485. 6735871

[B106] Neumann I. D. Veenema A. H. Beiderbeck D. I. (2010). Aggression and anxiety: social context and neurobiological links. Front. Behav. Neurosci. 4:12. 10.3389/fnbeh.2010.00012 20407578 PMC2854527

[B107] Nicolson T. J. Bellomo E. A. Wijesekara N. Loder M. K. Baldwin J. M. Gyulkhandanyan A. V.. (2009). Insulin storage and glucose homeostasis in mice null for the granule zinc transporter ZnT8 and studies of the type 2 diabetes-associated variants. Diabetes. 58, 2070–2083. 10.2337/db09-0551 19542200 PMC2731533

[B108] Ninh N. X. Thissen J. P. Maiter D. Adam E. Mulumba N. Ketelsiegers J. M. (1995). Reduced liver insulin-like growth factor-1 gene expression in young zinc-deprived rats in associated with a decrease in liver growth hormone (GH) receptors and serum GH-binding protein. J. Endocrinol. 144, 449–456. 10.1677/joe.0.1440449 7738469

[B109] Nowak G. Szewczyk B. Pilc A. (2005). Zinc and depression. An update. Pharmacol. Rep. 57, 713–718. 16382189

[B110] Nowak G. Szewczyk B. Wieronska J. M. Branski P. Palucha A. Pilc A.. (2003). Antidepressant-like effects of acute and chronic treatment with zinc in forced swim test and olfactory bulbectomy model in rats. Brain Res. Bull. 61, 159–164. 10.1016/s0361-9230(03)00104-7 12832002

[B111] Nriagu J. (2007). Zinc Deficiency in Human Health. School of Public Health, University of Michigan: Elsevier B.V., 1–7 Available online at: http://www.extranet.elsevier.com/homepage_about/mrwd/nvrn/Zinc%20Deficiency%20in%20Humans.pdf

[B112] Ohinata K. Takemoto M. Kawanago M. Fushimi S. Shirakawa H. Goto T.. (2009). Orally administered zinc increases food intake via vagal stimulation in rats. J. Nutr. 139, 611–616. 10.3945/jn.108.096370 19158231

[B113] Ohlsson C. Bengtsson B. A. Isaksson O. G. Andreassen T. T. Slootweg M. C. (1998). Growth hormone and bone. Endocr. Rev. 19, 55–79. 10.1210/edrv.19.1.0324 9494780

[B114] O’Kusky J. R. Ye P. D’Ercole A. J. (2000). Insulin-like growth factor-I promotes neurogenesis and synaptogenesis in the hippocampal dentate gyrus during postnatal development. J. Neurosci. 20, 8435–8442. 11069951 10.1523/JNEUROSCI.20-22-08435.2000 PMC6773150

[B115] Ott E. A. Smith W. H. Stob M. Beeson W. M. (1964). Zinc deficiency syndrome in the young lamb. J. Nutr. 82, 41–50. 14110939 10.1093/jn/82.1.41

[B116] Palmiter R. D. Cole T. B. Quaife C. J. Findley S. D. (1996). ZnT-3, a putative transporter of zinc into synaptic vesicles. Proc. Natl. Acad. Sci. U S A 93, 14934–14939. 10.1073/pnas.93.25.14934 8962159 PMC26240

[B117] Pan E. Zhang X. A. Huang Z. Krezel A. Zhao M. Tinberg C. E.. (2011). Vesicular zinc promotes presynaptic and inhibits postsynaptic long-term potentiation of mossy fiber-CA3 synapse. Neuron 71, 1116–1126. 10.1016/j.neuron.2011.07.019 21943607 PMC3184234

[B118] Pepersack T. Rotsaert P. Benoit F. Willems D. Fuss M. Bourdoux P.. (2001). Prevalence of zinc deficiency and its clinical relevance among hospitalised elderly. Arch. Gerontol. Geriatr. 33, 243–253. 10.1016/s0167-4943(01)00186-8 15374021

[B119] Peters D. P. (1978). Effects of prenatal nutritional deficiency on affiliation and aggression in rats. Physiol. Behav. 20, 359–362. 10.1016/0031-9384(78)90313-x 567810

[B120] Peters J. L. Dufner-Beattie J. Xu W. Geiser J. Lahner B. Salt D. E.. (2007). Targeting of the mouse Slc39a2 (Zip2) gene reveals highly cell-specific patterns of expression and unique functions in zinc, iron and calcium homeostasis. Genesis 45, 339–352. 10.1002/dvg.20297 17506078

[B121] Pfaender S. Grabrucker A. M. (2014). Characterization of biometal profiles in neurological disorders. Metallomics 6, 960–977. 10.1039/c4mt00008k 24643462

[B122] Piletz J. E. Ganschow R. E. (1978). Zinc deficiency in murine milk underlies expression of the lethal milk (lm) mutation. Science 199, 181–183. 10.1126/science.619449 619449

[B123] Prasad A. S. (2013). Discovery of human zinc deficiency: its impact on human health and disease. Adv. Nutr. 4, 176–190. 10.3945/an.112.003210 23493534 PMC3649098

[B124] Prasad A. S. Bao B. Beck F. W. J. Sarkar F. H. (2011). Zinc-suppressed inflammatory cytokines by induction of A20-medated inhibition of nuclear factor-kB. Nutrition 27, 816–823. 10.1016/j.nut.2010.08.010 21035309

[B125] Prasad A. S. Beck F. W. J. Bao B. Fitzgerald J. T. Snell D. C. Steinberg J. D.. (2007). Zinc supplementation decreases incidence of infections in the elderly: effect of zinc on generation of cytokines and oxidative stress. Am. J. Clin. Nutr. 85, 837–844. 17344507 10.1093/ajcn/85.3.837

[B126] Prasad A. S. Fitzgerald J. T. Hess J. W. Kaplan J. Pelen F. Dardenne M. (1993). Zinc deficiency in the elderly patients. Nutrition 9, 218–224. 8353362

[B127] Prasad A. S. Halsted J. A. Nadimi M. (1961). Syndrome of iron deficiency anemia, hepatosplenomegaly, hypogonadism, dwarfism and geophagia. Am. J. Med. 31, 532–546. 10.1016/0002-9343(61)90137-1 14488490

[B128] Prasad A. S. Miale A. Farid Z. Sandstead H. H. Schulert A. R. Darby W. J. (1963a). Biochemical studies on dwarfism, hypogonadism and anemia. Arch. Intern. Med. 111, 407–428. 10.1001/archinte.1963.03620280007003 13985936

[B129] Prasad A. S. Miale A. Farid Z. Schulert A. Sandstead H. H. (1963b). Zinc metabolism in patients with the syndrome of iron deficiency anemia, hepatosplenomegaly, dwarfism, and hypognadism. J. Lab. Clin. Med. 61, 537–549. 13985937

[B130] Riikonen R. Makkonen I. Vanhala R. Turpeinen U. Kuikka J. Kokki H. (2006). Cerebrospinal fluid insulin-like growth factors IGF-1 and IGF-2 in infantile autism. Dev. Med. Child Neurol. 48, 751–755. 10.1111/j.1469-8749.2006.tb01361.x 16904022

[B131] Roohani N. Hurrell R. Kelishadi R. Schulin R. (2013). Zinc and its importance for human health: an integrative review. J. Res. Med. Sci. 18, 144–157. 23914218 PMC3724376

[B132] Rosenkranz E. Prasad A. S. Rink L. (2011). “Immunobiology and hematology of zinc,” in Zinc in Human Health, ed Rink L. (Amsterdam, Netherlands: IOS Press), 195–233.

[B133] Russo A. J. (2011). Decreased zinc and increased copper in individuals with anxiety. Nutr. Metab. Insights 4, 1–5. 10.4137/nmi.s6349 23946656 PMC3738454

[B134] Russo A. J. Bazin A. P. Bigega R. Carlson R. S. Cole M. G. Contreras D. C.. (2012). Plasma copper and zinc concentration in individuals with autism correlate with selected symptom severity. Nutr. Metab. Insights 5, 41–47. 10.4137/nmi.s8761 23882147 PMC3698472

[B135] Saleptsis V. G. Labropoulos N. Halaris A. Angelopoulos N. V. Giannoukas A. D. (2011). Depression and atherosclerosis. Int. Angiol. 30, 97–104. 21427645

[B136] Sandstead H. H. Fosmire G. J. Halas E. S. Jacob R. A. Strobel D. A. Marks E. O. (1977). Zinc deficiency: effects on brain and behavior of rats and rhesus monkeys. Teratology 16, 229–234. 10.1002/tera.1420160219 412269

[B137] Sandstead H. H. Strobel D. A. Logan G. M. Marks E. O. Jacob R. A. (1978). Zinc deficiency in pregnant rhesus monkeys: effects on behavior of infants. Am. J. Clin. Nutr. 31, 844–849. 417617 10.1093/ajcn/31.5.844

[B138] Sazawal S. Bentley M. Black R. E. Dhingra P. George S. Bhan M. K. (1996). Effect of zinc supplementation on observed activity in low socioeconomic Indian preschool children. Pediatrics 98, 1132–1137. 8951265

[B139] Schoenthaler S. J. Bier I. D. (2000). The effect of vitamin-mineral supplementation on juvenile delinquency among American schoolchildren: a randomized, double-blind placebo-controlled trial. J. Altern. Complement. Med. 6, 7–17. 10.1089/acm.2000.6.7 10706231

[B140] Shankar A. H. Prasad A. S. (1998). Zinc and immune function: the biological basis of altered resistance to infection. Am. J. Clin. Nutr. 68, 447S–463S. 9701160 10.1093/ajcn/68.2.447S

[B141] Sindreu C. Palmiter R. D. Storm D. R. (2011). Zinc transporter ZnT-3 regulates presynaptic Erk1/2 signaling and hippocampus-dependent memory. Proc. Natl. Acad. Sci. U S A 108, 3366–3370. 10.1073/pnas.1019166108 21245308 PMC3044399

[B142] Sindreu C. Storm D. R. (2011). Modulation of neuronal signal transduction and memory formation by synaptic zinc. Front. Behav. Neurosci. 5:68. 10.3389/fnbeh.2011.00068 22084630 PMC3211062

[B143] Sivasubramanian K. N. Henkin R. I. (1978). Behavioral and dermatologic changes and low serum zinc and copper concentrations in two premature infants after parenteral alimentation. J. Pediatr. 93, 847–851. 10.1016/s0022-3476(78)81099-3 101647

[B144] Sladek R. Rocheleau G. Rung J. Dina C. Shen L. Serre D.. (2007). A genome-wide association study identifies novel risk loci for type 2 diabetes. Nature 445, 881–885. 10.1038/nature05616 17293876

[B145] Sowa-Kućma M. Legutko B. Szewczyk B. Novak K. Znojek P. Poleszak E.. (2008). Antidepressant-like activity of zinc: further behavioral and molecular evidence. J. Neural Transm. 115, 1621–1628. 10.1007/s00702-008-0115-7 18766297

[B146] Strobel D. A. Sandstead H. H. (1984). “Social and learning changes following prenatal or postnatal zinc deprivation in rhesus monkeys,” in The Neurobiology of Zinc. Part B: Deficiency, Toxicity and Pathology, eds Frederickson C. Howell G. A. Kasarskis E. J. (New York, NY: Alan R. Liss, Inc.), 121–138.

[B147] Suliman H. B. Abdelrahim A. I. Zakia A. M. Shommein A. M. (1988). Zinc deficiency in sheep: field cases. Trop. Anim. Health Prod. 20, 47–51. 10.1007/bf02239646 3354059

[B148] Tahmasebi Boroujeni S. Naghdi N. Shahbazi M. Farrokhi A. Bagherzadeh F. Kazemnejad A.. (2009). The effect of severe zinc deficiency and zinc supplement on spatial learning and memory. Biol. Trace Elem. Res. 130, 48–61. 10.1007/s12011-008-8312-7 19183867

[B149] Takeda A. Takefuta S. Okada S. Oku N. (2000). Relationship between brain zinc and transient learning impairment of adult rats fed zinc-deficient diet. Brain Res. 859, 352–357. 10.1016/s0006-8993(00)02027-8 10719084

[B150] Takeda A. Tamano H. (2009). Insight into zinc signaling from dietary zinc deficiency. Brain Res. Rev. 62, 33–44. 10.1016/j.brainresrev.2009.09.003 19747942

[B151] Takeda A. Tamano H. Kan F. Hanajima T. Yamada K. Oku N. (2008). Enhancement of social isolation-induced aggressive behavior of young mice by zinc deficiency. Life Sci. 82, 909–914. 10.1016/j.lfs.2008.02.005 18374363

[B152] Takeda A. Tamano H. Kan F. Itoh H. Oku N. (2007). Anxiety-like behavior of young rats after 2-week zinc deprivation. Behav. Brain Res. 177, 1–6. 10.1016/j.bbr.2006.11.023 17166602

[B153] Tamano H. Kan F. Kawamura M. Oku N. Takeda A. (2009). Behavior in the forced swim test and neurochemical changes in the hippocampus in young rats after 2-week zinc deprivation. Neurochem. Int. 55, 536–541. 10.1016/j.neuint.2009.05.011 19463882

[B154] Tassabehji N. M. Corniola R. S. Alshingiti A. Levenson C. W. (2008). Zinc deficiency induces depression-like symptoms in adult rats. Physiol. Behav. 95, 365–369. 10.1016/j.physbeh.2008.06.017 18655800

[B155] Todd W. R. Elvehjem C. A. Hart E. B. (1933). Zinc in the nutrition of the rat. Am. J. Physiol. 107, 146–156.

[B156] Wachs T. D. Bishry Z. Moussa W. Yunis F. McCabe G. Harrison G. (1995). Nutritional intake and context as predictors of cognition and adaptive behaviour of Egyptian school-age children. Int. J. Behav. Dev. 18, 425–450 10.1177/016502549501800303

[B157] Walsh W. J. Isaacson H. R. Rehman F. Hall A. (1997). Elevated blood copper/zinc ratios in assaultive young males. Physiol. Behav. 62, 327–329. 10.1016/s0031-9384(97)88988-3 9251975

[B158] Watanabe M. Tamano H. Kikuchi T. Takeda A. (2010). Susceptibility to stress in young rats after 2-week zinc deprivation. Neurochem. Int. 56, 410–416. 10.1016/j.neuint.2009.11.014 19931332

[B159] Wenzel H. J. Cole T. B. Born D. E. Schwartzkroin P. A. Palmiter R. D. (1997). Ultrastructural localization of zinc transporter-3 (ZnT-3) to synaptic vesicle membranes within mossy fiber boutons in the hippocampus of mouse and monkey. Proc. Natl. Acad. Sci. U S A 94, 12676–12681. 10.1073/pnas.94.23.12676 9356509 PMC25081

[B160] Wessells K. R. Brown K. H. (2012). Estimating the global prevalence of zinc deficiency: results based on zinc availability in national food supplies and the prevalence of stunting. PLoS One 7:e50568. 10.1371/journal.pone.0050568 23209782 PMC3510072

[B161] Whittle N. Hauschild M. Lubec G. Holmes A. Singewald N. (2010). Rescue of impaired fear extinction and normalization of cortico-amygdala circuit dysfunction in a genetic mouse model by dietary zinc restriction. J. Neurosci. 30, 13586–13596. 10.1523/JNEUROSCI.0849-10.2010 20943900 PMC3149823

[B162] Whittle N. Lubec G. Singewald N. (2009). Zinc deficiency induces enhanced depression-like behaviour and altered limbic activation reversed by antidepressant treatment in mice. Amino Acids 36, 147–158. 10.1007/s00726-008-0195-6 18975044

[B163] Willoughby J. L. Bowen C. N. (2014). Zinc deficiency and toxicity in pediatric practice. Curr. Opin. Pediatr. 26, 579–584. 10.1097/mop.0000000000000132 25029226

[B164] Wójcik J. Dudek D. Schlegel-Zawadzka M. Grabowska M. Marcinek A. Florek E.. (2006). Antepartum/postpartum depressive symptoms and serum zinc and magnesium levels. Pharmacol. Rep. 58, 571–576. 16963806

[B165] Xie X. Smart T. G. (1994). Modulation of long-term potentiation in rat hippocampal pyramidal neurons by zinc. Pflugers Arch. 427, 481–486. 10.1007/bf00374264 7971146

[B166] Yasuda H. Tsutsui T. (2013). Assessment of infantile mineral imbalances in autism spectrum disorders (ASDs). Int. J. Environ. Res. Public Health 10, 6027–6043. 10.3390/ijerph10116027 24284360 PMC3863885

[B167] Yasuda H. Yoshida K. Yasuda Y. Tsutsui T. (2011). Infantile zinc deficiency: association with autism spectrum disorders. Sci. Rep. 1:129. 10.1038/srep00129 22355646 PMC3216610

[B168] Yoshida M. Watanabe C. Horie K. Satoh M. Sawada M. Shimada A. (2005). Neurobehavioral changes in metallothionein-null mice prenatally exposed to mercury vapor. Toxicol. Lett. 155, 361–368. 10.1016/j.toxlet.2004.11.001 15649619

[B169] Yu X. Jin L. Zhang X. Yu X. (2013). Effects of maternal mild zinc deficiency and zinc supplementation in offspring on spatial memory and hippocampal neuronal ultrastructural changes. Nutrition 29, 457–461. 10.1016/j.nut.2012.09.002 23312766

